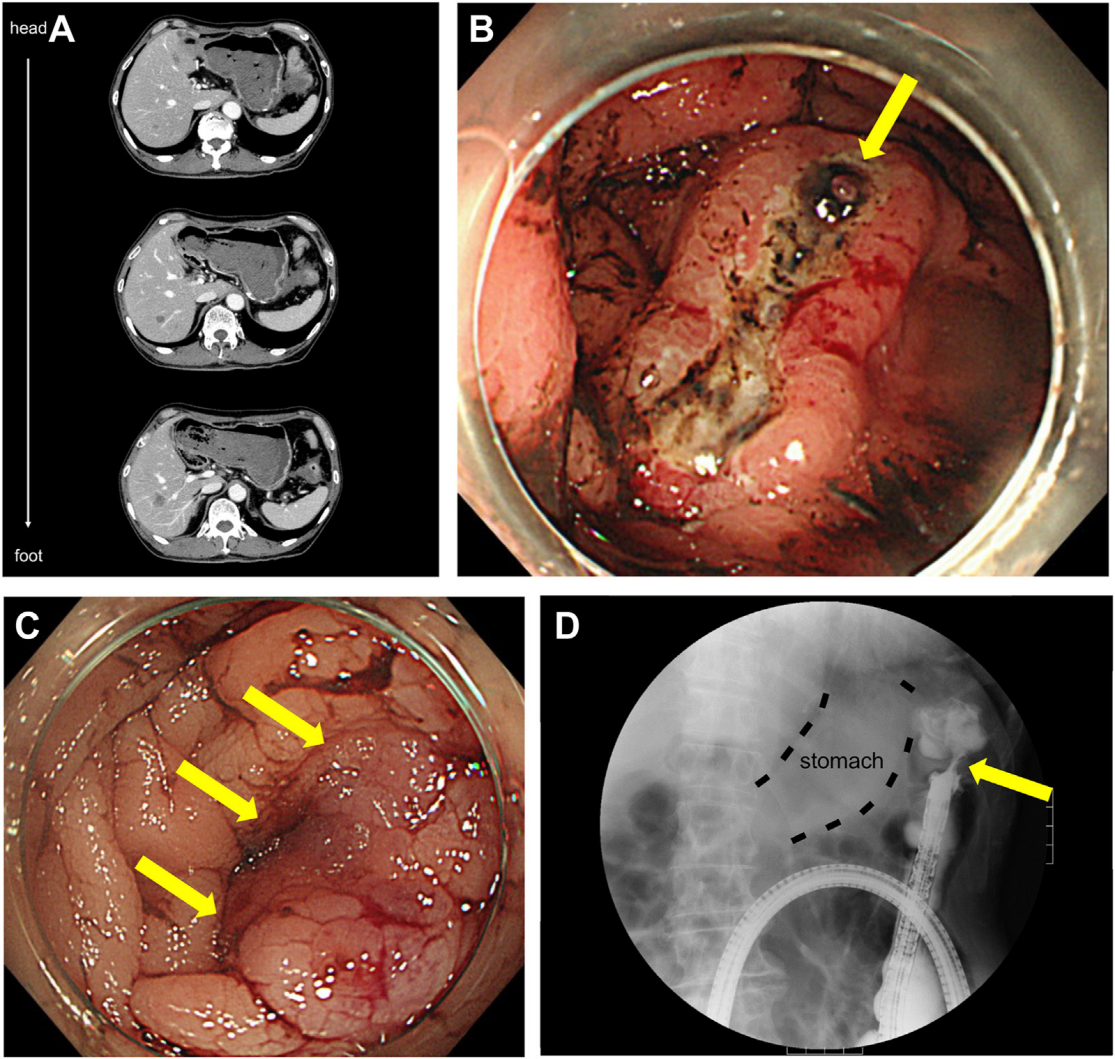# An Unusual Case of Gastrointestinal Bleeding; Which Is the Bleeding Origin, Upper or Lower Digestive Tract?

**DOI:** 10.1016/j.gastha.2023.05.006

**Published:** 2023-05-31

**Authors:** Kazuki Natsui, Masaki Maruyama, Shuji Terai

**Affiliations:** 1 Department of Gastroenterology, Kashiwazaki General Hospital and Medical Center, Kashiwazaki, Niigata, Japan; 2 Division of Gastroenterology and Hepatology, Graduate School of Medical and Dental Sciences, Niigata University, Chuo-Ku, Niigata, Japan

A 75-year-old man with no medical history presented to our emergency room with a massive bloody stool. His vital signs on arrival indicated shock, and blood tests showed decreased hemoglobin (9.2 g/dL; reference range, 12–16 g/dL). Computed tomography showed the stomach being full of high-density contents, a wall break in a part of the descending colon, a soft shadow from the wall break to the gastric side, and multiple liver tumors (Figure A, head side at the top). Urgent esophagogastroduodenoscopy showed massive blood clots and an ulcer of approximately 40 mm in size at the fornix (Figure B) with an exposed blood vessel (yellow arrow). Endoscopic coagulation was performed. Subsequently, urgent colonoscopy and lower gastrointestinal series showed a semicircular submucosal tumor (yellow arrows, Figure C) and scope-impassible stenosis (Figure D). Pathological findings for the gastric ulcer and colonic submucosal tumor were muc, tub1, and tub2. The patient was diagnosed with gastric cancer, and the histological type was poorly differentiated adenocarcinoma with colonic metastasis via peritoneal dissemination. He planned to undergo nivolumab/S-1/oxaliplatin chemotherapy.

As colonic metastasis of gastric cancer is extremely rare and presents difficult image finding to diagnose, intestinal findings in the presence of gastric cancer should be noted.

**Figure:**